# Are We Rational or Not? The Exploration of Voter Choices during the 2016 Presidential and Legislative Elections in Taiwan

**DOI:** 10.3389/fpsyg.2017.01762

**Published:** 2017-10-12

**Authors:** I-Ching Lee, Eva E. Chen, Nai-Shing Yen, Chia-Hung Tsai, Hsu-Po Cheng

**Affiliations:** ^1^Department of Psychology, National Chengchi University, Taipei, Taiwan; ^2^Research Center for Mind, Brain, and Learning, National Chengchi University, Taipei, Taiwan; ^3^Division of Social Science, Hong Kong University of Science and Technology, Hong Kong, Hong Kong; ^4^Election Study Center, National Chengchi University, Taipei, Taiwan

**Keywords:** explicit and implicit political party preferences, ethnic identification, significant others' opinions, voter intention and choices, path model testing

## Abstract

The decisions voters make—and whether those decisions are rational—have profound implications on the functionality of a democratic society. In this study, we delineated two criteria in evaluating voter rationality and weigh evidence of voter rationality versus irrationality. Furthermore, we compared models in two different elections in Taiwan to explore the reasons behind the irrational choices voters can make. Survey questions and an implicit association test (IAT) were administered prior to both elections among 197 voters in Taipei. These voters then reported their actual votes post-election. Model testing suggests that voters often are rational, but are more likely to make irrational choices in more important elections. Our findings indicate that voters generally aim to be diligent and to optimize their choices, even if they make less rational choices in the end. Further implications regarding elections and human rationality are discussed.

## Introduction

A well-functioning democracy relies upon its citizens to make rational decisions, especially when voting in elections (i.e., selecting the most capable candidates to achieve one's vision of an ideal society). Through their votes, individuals can help shape the leadership, laws, and policies of their society. However, voters do not always behave rationally. The importance of rational voting is clear when considering how numerous historical cases have resulted in democratic societies electing leaders that have caused great harm (e.g., the rise of Adolf Hitler and the National Socialist German Workers' [Nazi] Party via elections in Germany during the 1930s). While such tragedies may not have been purely the result of voter irrationality, they do stress the importance of ensuring rational voters.

Whether, when, and how humans are rational are issues that have long been debated in the social sciences. Human rationality generally refers to the ability to realize one's presumed goals (i.e., optimal choices; Tsebelis, 1990), although the criteria one should use to evaluate rationality remain debatable (Shapiro, 1969; Baker and Walter, 1975). On the one hand, objective measures can be implemented to evaluate voters' knowledge, understanding, and preferences with regards to the target issues (e.g., Campbell et al., 1964; Shapiro, 1969); doing so falls in line with the concept of *objective rationality* (Simon, 1982). For example, to evaluate candidates' capabilities to realize one's vision of an ideal society, one needs to have an understanding of the issues presented by different candidates and factor such an understanding in one's voting decision. The findings obtained using these measures suggest that voters are largely irrational because the reasons behind voters' decisions are often not specific to the target issues. On the other hand, researchers may use *subjective rationality* (Simon, 1982), examining whether voters' choices are determined by their intention to vote for the political candidates (e.g., Fishbein and Coombs, 1974; Granberg and Holmberg, 1990). If rationality is based on the consistency between individual attitudes and actual voting behavior, voters can be generally considered rational. Past scholars have highlighted the importance of evaluating human decisions and behavior through the lenses of subjective rationality, rather than imposing an overly narrow “definition of rationality—reasons are good when they are objectively good” (Boudon, 1989, p. 176).

To extend the concept of subjective rationality further, we argue that consistency between intention and behavior should not be the only criterion in judging one's rationality; the information with which individuals form their intentions should also be considered. One important determinant of one's intentions is the attitudes that one has toward other people and groups as well as toward events and situations. Psychologists have long studied the link between attitudes and behavior (e.g., Kraus, 1995), and found attitudes to be an important predictor for one's behavior [Baker and Walter, 1975; see the theory of planned behavior, Ajzen, 1991; the more recent reasoned action approach, Fishbein and Ajzen, 2010; and the MODE model (motivation and opportunity as determinants of the attitude-behavior relationship), Fazio and Olson, 2003]. However, previous work did not explicitly allude to human rationality (but see the discussion of the “reasoned action approach” in Fishbein and Ajzen, 2010). Further complicating matters, attitudes function at both explicit and implicit levels, such as in the MODE model (Fazio and Olson, 2003) or in the elaboration likelihood model (Petty and Cacioppo, 1986). While some researchers suggest that behaviors informed by implicit attitudes should be considered irrational (e.g., Zajonc, 1980; Glaser and Finn, 2013; Lodge and Taber, 2013), others have found behaviors informed by implicit attitudes to be a result of systematic processing and therefore rational (e.g., Cohen, 2003).

In light of the previous research on voter rationality (e.g., Fishbein and Coombs, 1974; Baker and Walter, 1975), we propose two key criteria in evaluating voter rationality. These two criteria help us integrate crucial elements in objective rationality (candidates' capabilities) and subjective rationality (intention to vote). Voters' decisions are rational if their voting behavior is based on (a) voters' intention (*intention-behavior consistency*), and if their intention is based on (b) voters' evaluations of the performance or capabilities of the candidate (*candidate evaluation*). Any decision not meeting the above two criteria would be considered irrational. We also explore distal factors that may be associated with voters' evaluations of the candidate, such as their preference for a particular political party. Party preference, though often considered a heuristic in voting, could be involved with systematic processing (Cohen, 2003). Thus, we evaluate whether voters' choices are informed by preferences of political parties, then candidate evaluations, which are mediated by voting intention. For example, if a Democratic voter votes for a Democratic candidate, even when he perceives the competing Republican candidate more capable of realizing his goals (e.g., how the society should function or reform) than the Democratic counterpart, this is not a rational choice but a result of the rigidity of partisanship.

Furthermore, we evaluate voter rationality by weighing the relative proportion of the rational choices versus irrational choices. We achieve this goal by estimating a model that compares variance explained by the paths meeting the two criteria with paths that do not. The current model is refined from previous research in evaluating the rationality of Taiwanese voters (Lee et al., 2016), which focused on three sets of predictors: *political party preference, ethnic identification*, and *the voting intention of significant others*. Research has identified political partisanship as one of the most robust factors in predicting voter intention and choices (e.g., Bartels, 2000; Hillygus and Jackman, 2003), both in Taiwan (Achen and Wang, 2017) and in other democratic societies (e.g., the U.S.; see Bartels, 2000). Additionally, ethnic identification has emerged as an important predictor in accounting for voters' choices in Taiwan (Achen and Wang, 2017). Finally, significant others have been found to sway voters' intention and choices (e.g., Huckheldt and Sprague, 1987; Beck et al., 2002): in Taiwan, social relationships are particularly valued (Hofstede, 1998, 2008).

To provide further background, we describe the political climate of Taiwan. The Taiwanese political parties can be classified into two camps, pan-Blue and pan-Green. The distinction of the two camps rests primarily on their views toward the People's Republic of China (PRC). The pan-Blue political parties, led by the Kuomintang Party (KMT), are perceived as being more supportive of closer ties with the PRC; while the pan-Green parties, led by the Democratic Progressive Party (DPP), are considered to be less supportive (Hsieh and Niou, 1996). Because attitudes toward Taiwan-PRC relations is a clear distinction of the two political party camps, ethnic identity issues (i.e., whether to identify as Taiwanese or Chinese) have dominated political discourse in Taiwan (Cheng, 2009). Voters who identify as Taiwanese are generally more supportive of a pan-Green candidate, while those who identify as Chinese are more supportive of a pan-Blue candidate (Wu and Tsui, 2010).

The previous study, which focused on the 2014 Taipei mayoral elections, found Taiwanese voters to be largely rational (Lee et al., 2016). The stated party preference (i.e., explicit party preference) and an interaction between explicit and implicit party preference (i.e., subconscious, more intuitive feelings toward one party) predicted voters' intention and, in turn, their voting choices. That is, the more voters favored a political party, the more likely they (a) intended to vote for a specific candidate from that party and (b) actually voted for that candidate in the election. Because of their awareness regarding their preference in selecting a candidate, both in intentionality and in actuality, voters were therefore considered rational.

However, there is evidence suggesting that voters can sometimes be irrational as well (Lee et al., 2016). Ethnic identification and perceived voting intention of significant others *did not* predict voting intention, but *did* predict actual voting choices in a real election. That is, when people (a) identified as Taiwanese rather than Chinese or (b) perceived that their significant others had a stronger intent to vote for a specific candidate, they were more likely to vote for the candidate with whom they believe to share their ethnic identification or the one consistent with their significant others' choice, even if they had shown no preference to vote for that particular candidate beforehand.

Although the previous study examined several important aspects of voter rationality—voter preference, intention, and choices—one crucial factor was not examined: voters' evaluation of specific candidates (Lee et al., 2016). Therefore, the present study focuses on whether voters compile information from different sources (e.g., political parties) to help them determine which candidate is more capable compared to the others. More specifically, we investigate whether voters evaluate candidates, make their voting decisions, and actually vote in a rational manner. Additionally, we aim to understand how and when voters become irrational.

To this end, we collected data in two elections, the presidential and legislative elections in Taiwan, which were held simultaneously on January 16, 2016. Presidential elections are generally believed to be more important and influential than congressional elections (e.g., Baker and Walter, 1975). Two sets of evidence support this relative importance assumption for the elections in Taiwan. In a study conducted with Taiwanese citizens in 2002, when asked whether the President or the Legislative Yuan (the unicameral legislature) has the power to decide the premier and ministers, 35.4% of the respondents reported that the president has sole power, whereas 12.4% reported that the legislature has sole power, with the remaining reported that both share the same amount of power (Wu and Wang, 2003). In addition, before the presidential and legislative elections were held at the same time (in the years 1995 to 2008), the turnout rates were visibly lower for the legislative elections (ranging from 58.7 to 68.3%) than the presidential elections (ranging from 76.3 to 82.7%; The Central Election Commission, 2017).

If voters lack motivation, they should rely on heuristics to make decisions (henceforth, irrational decisions) in the less important election; if voters fear that they are not capable of making the right decision, they should rely on others or other sources of information to make decisions in more important election. Previous research has shown that people are more likely to use heuristics or to give satisficing rather than optimizing responses (i.e., responses that are satisfactory but not the most appropriate versus the most appropriate responses; see Krosnick, 1999) if they lack sufficient motivation or interest (e.g., Maheswaran and Chaiken, 1991). Conversely, individuals may conform to others' decisions if they think that others make better decisions compared to themselves (Baron et al., 1996). If a lack of motivation or interest is the main reason for voters' irrational choices, it should be observed more frequently in the less important election, the legislative election. However, if voters intend to optimize their decisions but fear that they are not capable of doing so by themselves, they may conform to others' decision or use other source of information not directly related to their evaluations of the candidates, which result in irrational choices in the more important election, the presidential election.

In summary, the current research seeks to achieve three aims. First, we tested whether Taiwanese voters behaved consistently with their preference, candidate evaluation, as well as intention and choices in the 2016 presidential and legislative elections. Second, we weigh evidence of rationality (i.e., meeting the two criteria we set for rationality) and irrationality using a model testing. Third, if voters behaved irrationally, we investigated potential reasons behind their choices.

## Method

### Taiwan's elections in 2016

Data collection took place in Taipei, the capital of Taiwan, prior and after the elections for the 14th President and Vice President, as well as for the members of the 9th Legislative Yuan. In the legislative election, each voter can cast two ballots, one for the district-level legislative candidate and the other for the at-large party-list legislative candidate. Each tier (district-level, at-large party-list) is counted separately. Because voters do not vote directly for the at-large party-list candidates (instead, they vote for the political party represented by the candidate), we focus on the vote for the district-level candidates in the legislative election.

Although most of the electoral races were between the candidates of the leading pan-Blue and pan-Green parties (respectively, the KMT and the DPP), the DPP formed a coalition with several minor political parties as a strategic move to compete with the KMT for the legislative election in Taipei. As a result, although the DPP did not campaign explicitly for the candidates representing the minor parties, it was widely perceived as being supportive of these candidates.

### Ethics approval

The research was approved by the Research Ethics Committee, National Taiwan University (NTU-REC No. 201412ES001). Participants provided written informed consent before the study began.

### Participants

We recruited residents from all 12 districts in Taipei, Taiwan. We advertised our study using emails (i.e., panel participants from the Election Study Center of National Chengchi University), various social media platforms (i.e., Facebook and the Bulletin Board System, which are popular in Taiwan), and through personal connections. Although there is no minimum sample size for path analyses, we aimed for a sample size larger than 100 participants, in accordance with guidelines set by previous researchers (e.g., MacCallum, 1986; Hatcher and O'Rourke, 2013). We collected data from 274 respondents; of these respondents, 69 did not complete the IAT experiment and 8 were disqualified because they were from the ineligible electoral districts. In total, there were 197 participants (104 males, 85 females, and 8 participants who did not report their gender) who were eligible voters, and 76.6% were young adults (i.e., adults younger than 40 years of age). The majority of the respondents (68.5%) had previously voted in the 2012 presidential election.

### Procedure and materials

Approximately 1 month before the 2016 elections in Taipei, respondents were invited by email to complete an online survey. They were then asked to complete a longer survey and an implicit association test (IAT) at a public university in Taipei. The survey (see Supplementary Material) asked participants questions on their explicit political party preference; ethnic identity; evaluations of the candidates; and voting intentions for the next president, the next district-level legislator, and the party-list vote for the legislators. Participants also had to report the perceived voting intention of their significant others for the next president and the next district-level legislator. Candidate evaluations were compared among those who completed all the measures and those who missed at least one item (48.7% in presidential candidate items and 61.9% in legislative candidate items); the evaluations did not differ among the presidential candidates and legislative candidates (*p*s > 0.22). We replaced missing values with the treatment of expectation maximization.

Following previous research (Lee et al., 2016), we calculated explicit political party preference by contrasting the respondents' preference for the DPP over the KMT; that is, the higher the explicit political party preference scores, the more the respondents explicitly preferred the DPP over the KMT. Voting intention and perceived voting intention of significant others were calculated using the same rationale. Voting intention was measured by two items, one item for participant's intended candidate and one item for participant's certainty of such a decision. Intended candidate was coded as follows: 1 for Ing-Wen Tsai (the presidential candidate representing the DPP), −1 for the candidate Eric Chu (the presidential candidate representing the KMT), and 0 for all other candidates, including James Soong (a third-party presidential candidate). Similarly, the codes in the legislative election were coded as 1 for the DPP or DPP-endorsed candidates, −1 for the KMT or KMT-endorsed candidates, and 0 for other candidates. The strength of voting intention was calculated by multiplying the intended choice with the degree of certainty. Likewise, the perceived voting intention of significant others was estimated by multiplying the perceived significant other's intended candidate choice (coded the same way as for the participant's intended candidate) with the perceived certainty of the significant other. Higher scores indicate a preference for the DPP candidates over KMT candidates, as well as stronger certainty. We also measured how respondents evaluated the specific candidates (7 items for each presidential candidate, αs >0.89; 2 items for each legislative candidate, αs > 0.85), as well as whether they preferred male leaders, female leaders, or both.

Following the survey, respondents took a political party preferences IAT (for development and details of the test, please see Appendix A and Lee et al., 2016). We calculated the resulting D-scores so that higher D-scores indicated a stronger preference for the DPP (i.e., DPP = good). The order of the blocks did not affect the D-scores (*p* = 0.42), and therefore will not be further discussed.

One week after the conclusion of the 2016 elections, respondents were contacted again by phone to report their actual voting choice. Responses were coded the same way as was previously done for voting intention: 1 for the DPP or DPP-endorsed candidates, −1 for the KMT or KMT-endorsed candidates, and 0 for all other candidates.

## Results

Comparing the sample's reported votes with actual election results, there was a high consistency in reported presidential votes (60.3% sample vs. 56.1% population for the elected Tsai/Chen candidates) and party votes (the DPP getting the most votes both in the sample, 28.7% and 44.1% in the population, followed by the KMT 10.8% in the sample, 26.9% the population). Three out of eight legislative candidates received the most votes in the sample as in the actual election. There seems to have a bias in the sample in favor of the DPP and independent parties in legislative votes, probably due to the young and urban sample.

The respondents preferred the DPP party over the KMT party both explicitly and implicitly (*M*_*EXP*_ = 2.49 from a scale of −10 to 10, *SD* = 3.45; *M*_*IMP*_ = 0.14, *SD* = 0.62, mean scores different from the 0 point, *p*s < 0.01), and the two levels of attitudes were significantly correlated with one another, *r*_(*n* = 181)_ = 0.54, *p* < 0.001. Please see Appendix B for the complete set of analyses.

The majority of participants (70.1%) identified as only Taiwanese [*M* = 3.70, *SD* = 0.56, scores ranging from 1 (Chinese only) to 4 (Taiwanese only)]. Taiwanese identity was correlated with explicit and implicit party preferences, *r*s < 0.42, *p* < 0.001.

Respondents evaluated the DPP candidate Tsai (*M* = 2.78, *SD* = 0.56 on a scale of 1 to 4) more highly than the third-party candidate Soong *(M* = 2.52, *SD* = 0.61), while the KMT candidate Chu was the least favorable (*M* = 2.20, *SD* = 0.61), all pairwise contrasts at *p*s < 0.001 (*d* + s > 0.30). Similarly, respondents evaluated DPP-represented or endorsed legislative candidates more highly than KMT-represented candidates (*M* = 2.72, *SD* = 0.52 vs. *M* = 2.40, *SD* = 0.65, *d*+ = 0.34, *p* = 0.001).

**Figure 1:**
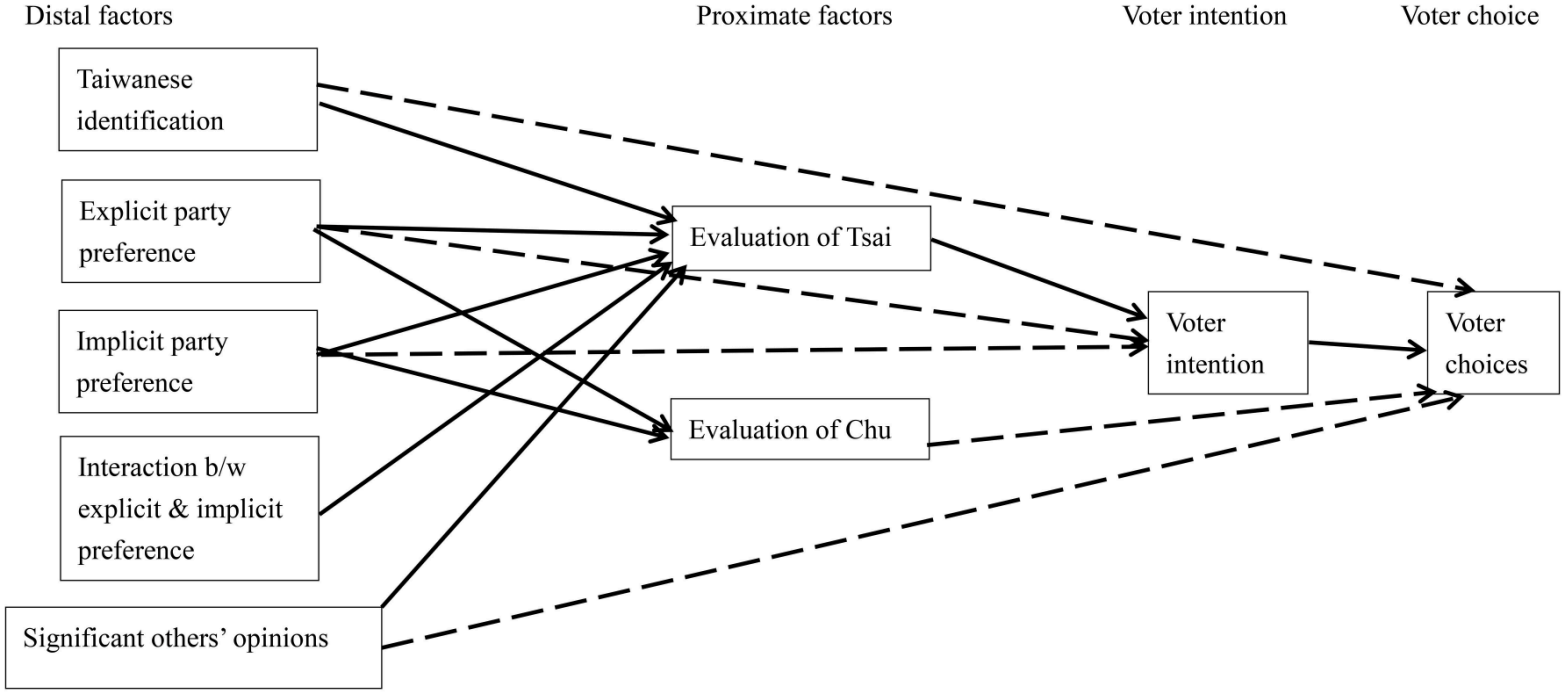
The path model of predictors on voter intention and choices in the presidential election. Participant gender and age were controlled for but not shown. Solid lines were those that meet the criteria of rationality. Dotted lines were not.

**Figure 2:**
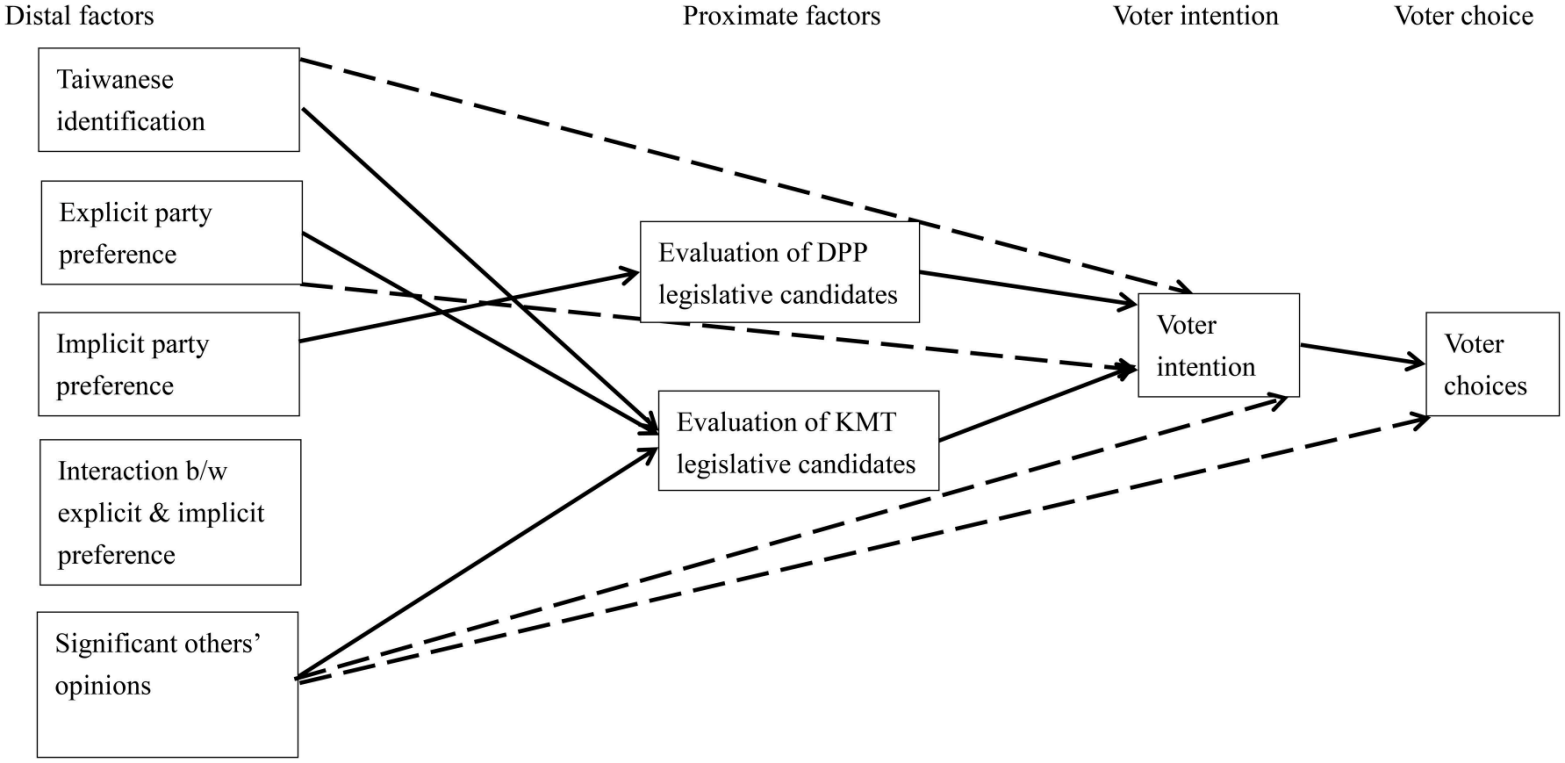
The path model of predictors on voter intention and choices in the legislative election. Participant gender and age were controlled for but not shown. Solid lines were those that meet the criteria of rationality. Dotted lines were not.

To examine voter rationality, we tested two path models in accounting for respondents' voting intention and voting choices (see Figures 1, 2). We first tested whether party preferences could predict evaluations of the specific candidates, which would in turn predict their voting intention and choices. In addition, we tested how two predictors—Taiwanese identification and significant others' opinions—may be associated with evaluations of the specific candidates, voting intention, and voting choices. Non-significant paths in both kinds of elections were dropped. Both models fitted the data well, χ^2^_(24)_ = 25.81, *p* = 0.36, for the presidential election and χ^2^_(30)_ = 29.97, *p* = 0.47, for the legislative election.

Extending from previous findings (Lee et al., 2016), rational choices best reflected voters' intention and behaviors because voting intention was a significant mediator in both types of elections (see standardized coefficients in Table 1 and significant indirect effects in Table 2). Explicit and implicit party preferences, as well as evaluations of specific candidates, significantly predicted their voting intention, and in turn, voting choices.

**Table 1:** Path models of voting intention and choices for presidential and legislative elections: Standardized coefficients.

**Paths**	**President**	**Legislative**
Explicit party preference 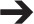 DPP candidate evaluation	0.41^***^	N/A
Implicit party preference 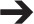 DPP candidate evaluation	0.21^***^	0.16^*^
An interaction b/w explicit and implicit party preference 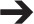 DPP candidate evaluation	0.11^*^	N/A
Taiwanese ID 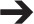 DPP candidate evaluation	0.13^*^	N/A
Significant others' opinions 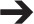 DPP candidate evaluation	0.15^**^	N/A
Age 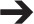 DPP candidate evaluation	−0.19^***^	N/A
Explicit party preference 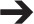 KMT candidate evaluation	−0.57^***^	−0.46^***^
Implicit party preference 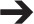 KMT candidate evaluation	−0.11^+^	N/A
An interaction b/w explicit and implicit party preference 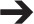 KMT candidate evaluation	N/A	N/A
Taiwanese ID 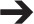 KMT candidate evaluation	N/A	0.18^**^
Significant others' opinions 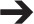 KMT candidate evaluation	N/A	−0.17^**^
Age 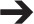 KMT candidate evaluation	0.22^***^	0.12^*^
Explicit party preference 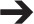 voting intention	0.28^***^	0.23^***^
Implicit party preference 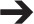 voting intention	0.21^***^	N/A
DPP candidate evaluation 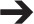 voting intention	0.48^***^	0.35^***^
KMT candidate evaluation 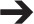 voting intention	N/A	−0.17^**^
Taiwanese ID 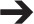 voting intention	N/A	0.12^*^
Significant others' opinion 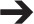 voting intention	N/A	0.33^***^
Voting intention 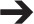 voting choice	0.42^***^	0.59^***^
Significant others' opinion 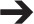 voting choice	0.13^*^	0.14^*^
KMT candidate evaluation 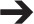 voting choice	−0.19^**^	N/A
Taiwanese ID 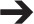 voter choice	0.15^**^	N/A
Age 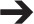 implicit party preference	−0.17^**^	−0.18^**^
Age 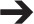 interaction b/w explicit and implicit party preferences	0.16^*^	0.18^*^
Age 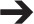 Taiwanese ID	−0.21^***^	−0.23^***^
Gender 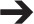 voting intention	−0.11^**^	N/A
Age 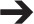 voting intention	N/A	−0.10^*^
Age 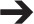 voting choice	N/A	−0.13^*^

+ p < 0.10;

* p < 0.05;

** p < 0.01;

*** *p < 0.001*.

**Table 2:** Standardized indirect effects and confidence intervals.

**Paths**	**President**	**Legislature**
Explicit party preference on voting intention	0.19 (0.14, 0.26)	0.08 (0.03, 0.13)
Explicit party preference on voting choices	0.31 (0.24, 0.39)	0.18 (0.12, 0.25)
Implicit party preference on voting intention	0.10 (0.05, 0.16)	0.06 (0.01, 0.10)
Implicit party preference on voting choices	0.15 (0.09, 0.22)	0.03 (0.008, 0.06)
Interaction b/w explicit and implicit party preference on voting intention	0.05 (0.01, 0.10)	0
Interaction b/w explicit and implicit party preference on voting choices	0.02 (0.01, 0.04)	0
KMT candidate evaluation on voting choices	0	−0.10 (−0.16, −0.04)
DPP candidate evaluation on voting choices	0.20 (0.14, 0.28)	0.21 (0.14, 0.27)
Significant others' opinion on voting intention	0.07 (0.03, 0.13)	0.03 (0.01, 0.06)
Significant others' opinion on voting choices	0.03 (0.01, 0.06)	0.21 (0.15, 0.29)

Furthermore, the evidence suggests that when the elections are more important (i.e., the presidential election, compared to the legislative election) to the voters, they become less systematic. As can be seen in Table 3, 22.8% of the total effects in voting choices met all our criteria in rationality for the presidential election, in contrast with the 36.3% of the total effects observed in the legislative election. Furthermore, candidates' evaluations met our criteria for rationality in legislative election (100%), compared with 50.9% of the variance in presidential election.

**Table 3:** Effects accounting for rational and irrational choices in the two elections.

**Predictors**	**Presidential election**		**Legislative election**	
	**Rational**	**Irrational**	**Rational**	**Irrational**
Evaluation of candidates	0.201	0.194	0.306	0
	(50.9%)	(49.1%)	(100%)	(0.0%)
Explicit attitudes	0.082^a^	0.229^b^	0.046^a^	0.137
	(26.4%)	(73.6%)	(25.1%)	(74.9%)
Implicit attitudes	0.042^a^	0.108^b^	0.032^a^	0
	(28.0%)	(72.0%)	(100%)	(0.0%)
Dual attitudes	0.022^a^	0	0	0
	(100.0%)	(0.0%)	(0.0%)	(0.0%)
Taiwanese identification	0.025^a^	0.152	0.018^a^	0.072
	(14.1%)	(85.9%)	(20.0%)	(80.0%)
Significant others' intention	0.030^a^	0.128	0.017^a^	0.329
	(19.0%)	(81.0%)	(4.9%)	(95.1%)
Total effects	0.201	0.679	0.306	0.538
	(22.8%)	(77.2%)	(36.3%)	(63.7%)

*The indirect effects were estimated by multiplying the standardized coefficients of the paths*.

a *Included in the evaluation of candidates*.

b *Some variances were included in the evaluation of candidates*.

Consistent with our hypothesis that voters may rely on heuristics when the consequence of the election is important, respondents were more likely to rely on heuristics such as Taiwanese identification to cast their votes (see Tables 1, 3). After accounting for the indirect effects, Taiwanese identification was associated significantly with one's voting choices in the presidential election, but not in the legislative election.

## Discussion

Are voters rational or irrational? On the one hand, research based on the objective definition of rationality suggests that voters are largely irrational because voters have been shown to lack sufficient knowledge about candidates in elections (Miller and Stokes, 1963); to form opinions on fabricated issues (Sturgis and Smith, 2010), to believe that their individual votes are representative of like-minded voters (i.e., the voters' illusion; Acevedo and Kruger, 2004), and to be affected by irrelevant events (e.g., outcome of sports events; Healy et al., 2010). On the other hand, research based on the subjective definition of rationality suggests that voters are largely rational because voters have shown great consistency in their attitudes and choices (e.g., Fishbein and Coombs, 1974), especially among those who considered the issues at hand important (Krosnick, 1988).

We offer an innovative framework in readdressing the question of voter rationality. We define rationality by voters' intention (*intention-behavior consistency*) and voters' evaluations of the performance or capabilities of the candidate (*candidate evaluation*). Thus, instead of using a specific set of variables to define voter rationality (e.g., accuracy in issue knowledge), we delineated a model specifying causal paths from distal factors (explicit and implicit party attitudes) and proximate factors (candidate evaluations) to voter intention and choices, evaluating the evidence holistically. Our framework helps us to draw a conclusion that differs substantially from a previous study conducted by Baker and Walter (1975). In the older study, the authors compared congressional and presidential voting in Wyoming, U.S.A., and concluded that voters are more rational when voting for the next president. Specifically, they found that in regression models, the preselected variables explain 44% of the variance in voting choices for the president, but only 28% for legislators. In addition, they found that the association between campaign issues and actual vote is stronger in the presidential than in the congressional elections (*r* = −0.65 vs. *r* = −0.52).

Here, we utilize modern analyses (i.e., path model) to extend the findings from previous work (Baker and Walter, 1975) and examine voter rationality in the Taiwanese presidential and legislative elections. No single variable in our research is predefined to reflect voter rationality. Instead, we evaluate the evidence holistically by considering voters rational when they used different criteria to evaluate the candidates, select the best candidate, and vote for that candidate. Evaluating the evidence holistically, we found that voters are partially rational, at least in the legislative election. In the more important presidential election, voters may have tried to optimize their performance, but inadvertently relied more on peripheral cues (e.g., their identification as Taiwanese). Our findings suggest that to improve voter rationality, summaries of the issues and fact-checking beliefs could be produced by neutral organizations in order to encourage more rational, rather than heuristic, voting behavior.

Unexpectedly, the association between explicit party preference and DPP candidate evaluation was not significant. This non-significant association may be due to the fact that most of the legislative candidates (six out of eight candidates) endorsed by the DPP in Taipei city were not from the DPP but were from minority parties considered to be pan-Green and supported by the DPP. The other unexpected finding was an inconsistency between our present study and our previous study. While the present study found that implicit party preference predicted both the evaluations of specific candidates and voting intention for the president (though not for the legislators), the previous study (Lee et al., 2016) found that explicit party preference was the key predictor on voter intention and choice in the Taipei mayoral elections. This discrepancy could be due to two factors. First, when evaluating candidates at the presidential level, political and social issues, (such as cross-strait relations, the economy, government pensions, and education) may have become so complicated that individuals defaulted to implicit party preference to form evaluations and decisions. Second, individuals may rely more on implicit party preference to form evaluations and decisions in elections of a larger scale and of higher importance relative to elections of a smaller scale and of lower importance. Future research is needed to systematically examine the impact of election scope on the predictability of implicit party attitudes on voting intention.

Taken together, our research provides evidence that the criteria we set for rationality—candidate evaluation and intention-behavior consistency—can be readily applied to examining voter decisions in two major democratic elections. Voters are largely rational in their voting decisions, though the degree of rationality depends the type and scope of election. In addition to a more systematic processing of relevant information before making their voting decisions, the explicit and implicit preferences of individuals exert varying levels of influence in their decision-making. Given the crucial role elections play in a functioning democratic society, gaining deeper insight into how rational and irrational human beings can become, before and when they cast their votes, is essential.

## Author contributions

IL, EC, NY, and CT developed the study concept and designed the study. IL, EC, NY, CT, and HC helped created materials and collected data. IL performed data analysis and interpretation and drafted the manuscript. EC, NY, and CT provided critical revisions.

### Conflict of interest statement

The authors declare that the research was conducted in the absence of any commercial or financial relationships that could be construed as a potential conflict of interest.
