# Supplementary material for: Are We Rational or Not? The Exploration of Voter Choices during the 2016 Presidential and Legislative Elections in Taiwan
Source: Front Psychol. 2017 Oct 12;8:1762. doi: 10.3389/fpsyg.2017.01762 (PMC5643908; doi:10.3389/fpsyg.2017.01762)
Supplement: Supplementary file 1 [file SupplementaryMaterial.DOCX]

| **Constructs** | **Example items** |
| --- | --- |
| **Political party ID (2 items)** | 1. Currently, there are the following major parties in our country: the Kuomintang, the Democratic People’s Party, the People First Party, the New Party, and the Taiwan Solidarity Union Party. Which party are you inclined to support? |
|  | 1. To what degree are you inclined to support your chosen party? |
| **Explicit political party preference** | If 0 represents “strongly dislike” and 10 represents “strongly like,” how would you score the two main national parties? |
| **(2 items)^a,b^** | 1. KMT: ______ |
|  | 1. DPP: _____ |
| **Candidate evaluations (presidential)** | 1. Capabilities in handling cross-Strait issues 2. Capabilities in economic development 3. Capabilities in having connections with the general public 4. Being honest and uncorrupted 5. Capabilities in deal with social security problems 6. Leadership capabilities 7. Capabilities in understanding the needs of the general public |
| **Candidate evaluations (congressional)** | 1. Capabilities in handling cross-Strait issues 2. Capabilities in economic development |
| **Ethnic ID (one item)^c^** | In our society, some people identify themselves as Chinese, and some people identify themselves as Taiwanese. How would you identify yourself? |
| **Voting intention (2 items)^a^** | 1. If you will vote in the upcoming elections, which mayoral candidate are you more likely to vote for? |
|  | 1. How sure are you about your voting intent? |
| **Perceived voting intention of significant others** | 1. Please think about a close family member or friend who has the most impact on you in terms of politics. Which mayoral candidate is the person likely to vote for? |
| **(2 items)^a^** | 1. How sure is your family member or friend about their voting intent? |
