# Supplementary material for: Are We Rational or Not? The Exploration of Voter Choices during the 2016 Presidential and Legislative Elections in Taiwan
Source: Front Psychol. 2017 Oct 12;8:1762. doi: 10.3389/fpsyg.2017.01762 (PMC5643908; doi:10.3389/fpsyg.2017.01762)
Supplement: Supplementary file 2 [file AppendixAB.docx]

Appendix

|  | Implicit preference^a^ | Dual preference | Tsai/DPP | Chu/KMT | TWID | Significant  Others’ | Vote intention | Vote choices |
| --- | --- | --- | --- | --- | --- | --- | --- | --- |
| Explicit preference^a^ | .54*** | -.13* | .61*** | -.63*** | .37*** | .45*** | .68*** | .53*** |
| Implicit preference^a^ | .54*** | -.20** | .55*** | -.46*** | .42*** | .39*** | .62*** | .50*** |
| Dual preference | -.13+ | -.20** | -.05 | .10 | -.18** | -.15* | -.11 | -.20** |
| Tsai/DPP | .15* | .16* | -.04 | -.58*** | .43*** | .45*** | .77*** | .54*** |
| Chu/KMT | -.61*** | -.46*** | .06 | -.14+ | -.36*** | -.36*** | -.58*** | -.53*** |
| Taiwanese identification | .37*** | .42*** | -.18* | .00 | -.40*** | .24*** | .39*** | .41*** |
| Significant others’ opinions | .45*** | .39*** | -.15* | .03 | -.35*** | .24*** | .48*** | .43*** |
| Vote intention | .68*** | .62*** | -.11 | .31*** | -.56*** | .39*** | .48*** | .65*** |
| Vote choices | .53*** | .50*** | -.20** | .15* | -.49*** | .41*** | .43*** | .65*** |
| Presidential Mean (SD.) | 0.00 (1.00) | 0.53 (0.89) | 2.78 (0.56) | 2.20 (0.61) | 3.70 (0.54) | 0.51 (8.18) | 3.88 (6.37) | 0.57 (0.66) |
| Congressional Mean (SD.) | 0.00 (1.00) | 0.53 (0.89) | 2.72 (0.52) | 2.40 (0.65) | 3.70 (0.54) | 0.51 (8.18) | 3.88 (6.37) | 0.57 (0.66) |

Note. +: *p* < .10; *: *p* < .05; **: *p* < .01; ***: *p* < .001. Correlations of presidential election were shown upper diagonal; correlations of congressional election were shown lower diagonal. a. Standardized scores.
